# The Role of *clbF* in the Pathogenicity of Avian Pathogenic *Escherichia coli*

**DOI:** 10.3390/vetsci12080727

**Published:** 2025-08-01

**Authors:** Meng Wu, Haitao Wu, Ling Li, Pan Hao, Peili Wang

**Affiliations:** 1 School of Animal Pharmaceutical, Jiangsu Agri-Animal Husbandry Vocational College, Taizhou 225300, China; 2015020301@jsahvc.edu.cn; 2 School of Pet Technology, Jiangsu Agri-Animal Husbandry Vocational College, Taizhou 225300, China; 2015020299@jsahvc.edu.cn (H.W.); 2007020099@jsahvc.edu.cn (L.L.); 3 School of Animal Husbandry and Veterinary Medicine, Jiangsu Vocational College of Agriculture and Forestry, Jurong 212400, China; panhao@jsafc.edu.cn; 4 The Third Clinical Medical College, Nanjing University of Chinese Medicine, 138 Xianlin Rd, Nanjing 210023, China; 5 Jiangsu Province Academy of Traditional Chinese Medicine, Nanjing 210028, China

**Keywords:** colibactin, avian pathogenic *Escherichia coli*, *clbF*, pathogenicity

## Abstract

The genotoxin colibactin, a complex secondary metabolite, targets eukaryotic cell cycle machinery and contributes to neonatal sepsis and meningitis. Avian pathogenic *Escherichia coli* (APEC) XM, which produces this genotoxin, is an agent of poultry diseases with zoonotic potential. In this study, we confirmed that *clbF* was necessary for the APEC XM strain to produce colibactin, but it did not affect the growth, adhesion, or invasion of cells. Deletion of *clbF* substantially diminished both virulence and systemic dissemination, but it also changed the gene expression of the antiserum survival factor, adherence and invasion, iron acquisition genes, and the secretion system. In conclusion, *clbF* is necessary for the synthesis of the genotoxin colibactin and affects the development of APEC meningitis in mice.

## Simple Summary

Avian colibacillosis is an infection caused by Avian pathogenic *Escherichia coli* (APEC) resulting in high morbidity, high mortality, and significant economic losses. The genotoxin colibactin has been identified as an important virulence factor for *Escherichia coli*, contributing to pathogenicity in various extraintestinal infections. In this study, deletion of *clbF* abolished colibactin production in APEC XM without impairing bacterial growth. Crucially, *clbF* deficiency significantly attenuated the strain’s virulence and altered expression of key virulence-associated genes, establishing its essential role in APEC pathogenicity. Despite current limitations, such as the lack of in-depth mechanistic studies on subsequent in vivo infection, this study provides a crucial first step in understanding *clbF*’s impact on *Escherichia coli* pathogenicity. Future research should focus on competition between colibactin-producing and non-producing strains, as well as infection dynamics in *clbF*-deficient mutants during in vivo host colonization, to elucidate the precise role of *clbF* in systemic infection.

## 1. Introduction

Avian colibacillosis is an infection caused by Avian pathogenic *Escherichia coli* (APEC) resulting in high morbidity, high mortality, and significant economic losses. APEC belongs to extraintestinal pathogenic *Escherichia coli* (ExPEC) [1], which shares similarities with neonatal meningitis *Escherichia coli* (NMEC) [2]. APEC and ExPEC share common virulence genes, such as *iucC*, *hlyA*, *iss*, and *ompT* [3]. APEC isolates obtained from poultry meningitis cases demonstrate the ability to cause urinary tract infection and meningitis in mouse and rat models [1,4]. Several studies suggest APEC may be a potential foodborne zoonotic pathogen as well as a reservoir for urinary or nervous system infections in humans [1,3].

Colibactin was initially identified in a Finnish neonatal meningitis-sepsis isolate (IHE3034) [5]. This genotoxic compound is produced by bacteria harboring the polyketide synthase (*pks*) genomic island, such as *Escherichia coli*, *Citrobacter koseri*, and *Klebsiella pneumoniae*. Colibactin induces DNA double-strand breaks and genomic damage in eukaryotic and prokaryotic cells [5]. These genotoxic effects trigger cell cycle arrest and, through activation of the bacterial response of DNA damages, prophage lytic development [6]. Colibactin is a suspected pro-carcinogenic factor for colorectal cancer [7,8] and has been identified as an important virulence factor for ExPEC, contributing to pathogenicity in various extraintestinal infections [9,10]. Furthermore, *pks^+^* strains can be categorized into three ecological types: pathogenic, commensal (fecal), and probiotic [11].

Nineteen genes (*clbA* to *clbS*) comprise the 54 kb genomic island known as *pks* island. In colibactin biosynthesis, the polyketide synthase (PKS) and nonribosomal peptide synthetase (NRPS) assembly line uniquely generates a rare PKS extender unit (AM-ACP), which is critical for constructing genotoxic metabolites [12]. In 2015, Piel and colleagues demonstrated that ClbD and ClbF exhibit homology to ZmaG and ZmaI in zwittermicin biosynthesis and are responsible for producing the AM-ACP extender unit [13]. However, whether ClbF possesses additional roles in colibactin biosynthesis remains unclear.

In this study, we used APEC XM (O2:K1), which was isolated from a meningitis and sepsis duck. This strain induces severe meningitis and sepsis in ducklings, chicks, young mice, and newborn rats [14,15,16]. Previous studies have examined the roles of *clbA*, *clbG*, and *clbH* in disease pathogenesis [17,18,19]. However, whether *clbF* is one of the key virulence factors contributing to meningitis caused by APEC XM has not yet been reported. Here, we deleted the *clbF* gene in APEC XM and elucidated how *clbF*-mediated colibactin production contributes to APEC XM pathogenicity. We assessed invasion and adhesion capacity, LD_50_ infection experiment, the bacterial loads of various organs, and the expression of virulence factors associated with survival, adhesion/invasion, iron uptake, and secretion system genes. These findings will elucidate the role of *clbF* in APEC pathogenicity.

## 2. Materials and Methods

### 2.1. Ethics Statement

The animal experiments followed the National Institute of Health guidelines for the ethical use of animals in China. All procedures were approved by the Animal Care and Ethics Committee of Jiangsu Agri-animal Husbandry Vocational College (JSAHVC-2025-78). Four-week-old male ICR mice were housed under standard conditions (12 h light/12 h dark). All procedures used isoflurane anesthesia to ensure the animals’ welfare.

### 2.2. Bacterial Strains, Plasmids, and Culture Conditions

The bacterial strains and plasmids used in this study are listed in Table S1. The APEC XM strain (O2:K1) and the plasmids (pKD3, pKD46, pCP20, and pBR322) were kindly donated by Prof. Guoqiang Zhu at Yangzhou University. The APEC XM strain was isolated from the brain of a duck with septicemia and meningitis. The deletion mutant and complemented mutant of *clbF* were generated from the APEC XM. All bacterial strains were cultured aerobically in Luria–Bertani (LB) broth at 37 °C with 180 rpm shaking. Strains carrying the temperature-sensitive plasmid pCP20 or pKD46 were grown at 30 °C. The LB broth or LB agar plates (Beyotime, Shanghai, China, ST156, ST158) contained ampicillin (Amp+, 100 μg/mL) (Sangon Biotech, Shanghai, China, B541011) and/or chloramphenicol (Cm+, 34 μg/mL) (Sangon Biotech, Shanghai, China, B541015) as required.

### 2.3. Construction of the clbF Deletion Mutant and the Complemented Mutant

All primers used for mutant generation are provided in Table S1. The *clbF* deletion mutant was generated using the λ Red recombination system, as previously described [20]. The full-length *clbF* sequence was cloned into plasmid pBR322 using the primer pBRclbF-F and pBRclbF-R. Subsequently, the recombinant plasmid pBR322-clbF was transformed into the *clbF* deletion mutant to construct the complemented strain. Mutant verification was performed by PCR and DNA sequencing for both the deletion mutant (APEC Δ*clbF*) and complemented strain (APEC Δc*lbF*/p*clbF*). The detail method of electroporate and PCR are provided in the Supplementary Materials.

### 2.4. Growth Test

To determine the growth rates of the APEC XM (WT), APEC Δ*clbF*, and APEC Δ*clbF* p*clbF* strains, all the bacteria were grown at 37 °C in 150 mL LB under continuous agitation (180 rpm). The optical density at 600 nm of the bacterial cultures was measured hourly for 20 h by a microplate reader (BioTek ELx800, Winooski, VT, USA). The growth curve experiment was performed with three biological replicates.

### 2.5. Mouse Brain Microvascular Endothelial Cells (bEnd.3) Adhesion and Invasion Assay

Cell adhesion assays were carried out as described previously [17]. The bEnd.3 cell line was purchased from the National Collection of Authenticated Cell Cultures (SCSP-5267, Shanghai, China). Cells were cultured in 6-well plates. Bacterial strains (log-phase cultures) underwent two phosphate-buffered saline (PBS, Beyotime, Shanghai, China, ST448) washes and were resuspended in Dulbecco’s modified eagle medium (DMEM, Gibco, Carlsbad, CA, USA, 12634010) prior to assays. Monolayers at 90% confluency were infected at multiplicity of infection (MOI) 100 for 3 h. Unbound bacteria were removed by three sterile PBS washes. For invasion assay, infected cells were incubated in DMEM with 10% fetal bovine serum (FBS, Gibco, Carlsbad, CA, USA, A5256701) containing gentamicin (100 μg/mL, Sangon Biotech, Shanghai, China, A428430) for 1 h to eliminate extracellular bacteria. Cells were then lysed with 0.5% Triton X-100 (37 °C, 30 min, Beyotime, Shanghai, China, ST1723). Lysates underwent 10-fold serial dilution, and viable bacteria were enumerated on LB agar plates.

### 2.6. Colibactin Cytotoxicity Assays

The bEnd.3 cells (75% confluent) were infected (MOI = 100) with the respective strains. At 4 h post-infection (hpi), the monolayers were PBS-washed and maintained in DMEM/10% FBS with gentamicin (100 μg/mL). To quantify colibactin-induced DNA double-strand breaks (DSBs), γH2AX expression was analyzed via immunofluorescence at 72 hpi. Infected cells were fixed, permeabilized (0.1% Triton X-100, 20 min), and processed using immunofluorescence following an established protocol [17]. For colibactin-induced megalocytosis, infected cells were fixed (4% paraformaldehyde fix solution, Beyotime, Shanghai, China, P0099) at 72 hpi and stained (0.1% methylene blue, 20 min). Cytotoxicity was quantified by the absorbance values of each group at 630 nm by a microplate reader (BioTek ELx800, Winooski, VT, USA).

### 2.7. Mouse Infection Model Experiment

The 50% lethal dose (LD_50_) was performed as described previously [16] to assess virulence in mouse models. Groups of five 4-week-old ICR mice were injected intramuscularly with bacterial suspensions containing 10^4^–10^7^ CFU of bacteria in sterile PBS, while control groups were administered PBS alone. For bacterial dissemination analysis, groups of six 4-week-old ICR mice were intraperitoneally injected with 10^7^ CFU each strain in 100 μL saline or an equal volume of sterile saline. The brain, spleen, and blood were collected from each euthanized mouse after 12 h post-infection. Bacteria were isolated from the brains, spleens, and blood and plated on MacConkey agar using 10-fold serial dilutions.

### 2.8. RNA Extraction and Real-Time Quantitative Polymerase Chain Reaction (RT-qPCR)

The total RNA was extracted from log-phase bacterial cultures using TRIzol reagent (Invitrogen, Carlsbad, CA, USA, 15596026CN) as per the manufacturer’s instructions. The high-quality RNA was converted into cDNA using the PrimeScript RT reagent Kit with gDNA Eraser (Takara, Tokyo, Japan, RR047A). RT-qPCR was performed using the CFX CONNECT Real-time PCR system (Bio-Rad, Hercules, CA, USA,) with ChamQ SYBR Master Mix (Vazyme Biotech, Nanjing, China, Q711-02/03), following this protocol: 95 °C for 10 min; 40 cycles of 95 °C for 30 s, 60 °C for 30 s, and 72 °C for 30 s; final extension at 72 °C for 10 min. Based on transcriptome data from APEC XM infection of bEnd.3 cells [21], 14 virulence-related genes across four categories were selected for detection. There were antiserum survival factor genes (*ompA*, *ompW,* and *iss*), adherence and invasion genes (*ibea*, *fimH*, *tsh*, and *fimA*), iron acquisition genes (*ireA*, *fyuA*, and *ChuA*), and secretion system genes (*Sec ABGY*). Primers for target genes are listed in Table S1. Gene expression was normalized to *gapA* and calculated via the 2^−ΔΔCt^ method.

### 2.9. Statistical Analysis

Data were analyzed with GraphPad Prism 9.0 software (San Diego, CA, USA) using one-way ANOVA for multiple comparisons. Two different levels of significance were defined: *p* < 0.05 (indicated by *) and *p* < 0.01 (indicated by **).

## 3. Results

### 3.1. Deletion of clbF Did Not Affect Growth Kinetics, Adhesion, or Invasion

**Figure 1:**
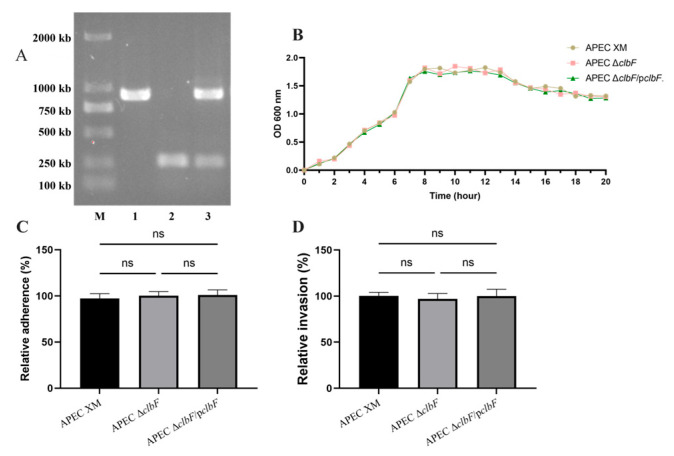
Verification of mutants and the ability of reproduction, adherence, and invasion. (**A**). Identification of mutants by PCR. The three strains, APEC, APEC Δ*clbF*, and APEC Δ*clbF*/p*clbF*, were amplified with primer P1/P2. (Lane M, DNA molecular size marker; Lane 1, APEC XM; Lane 2, APEC Δ*clbF*; and Lane 3, APEC Δ*clbF*/p*clbF*) (**B**). Growth curves analysis. The three strains, APEC, APEC Δ*clbF*, and APEC Δ*clbF*/p*clbF*, were grown in LB broth at 37 °C and the OD_600_ values were measured every 1 h over a period of 20 h. Each curve represents the averages of three independent experiments. Effects of *clbF* on adherence to (**C**) and invasion (**D**) of bEnd.3 cells (MOI = 100). All assays were carried out in triplicate (ns, *p* > 0.05).

The successful construction of the single gene deletion mutant APEC Δ*clbF* and the complemented strain APEC Δ*clbF*/p*clbF* were confirmed by PCR (Figure 1A). DNA sequencing verified genetic stability in both strains through 30 passages without mutations. APEC Δc*lbF* and APEC Δ*clbF*/pc*lbF* grew at a very similar rate to the APEC XM strain in the broth media. (Figure 1B). Multiple studies have supported that cell–cell contact is necessary for colibactin genotoxicity [22]. WT, Δ*clb*F, and complemented strains exhibited similar adherence to and invasion of bEnd.3 cells after 3 h infection (Figure 1C,D). These results suggest that *clbF* does not significantly contribute to growth, adhesion, or invasion of cell infection.

### 3.2. clbF Affects the Colibactin Production

**Figure 2:**
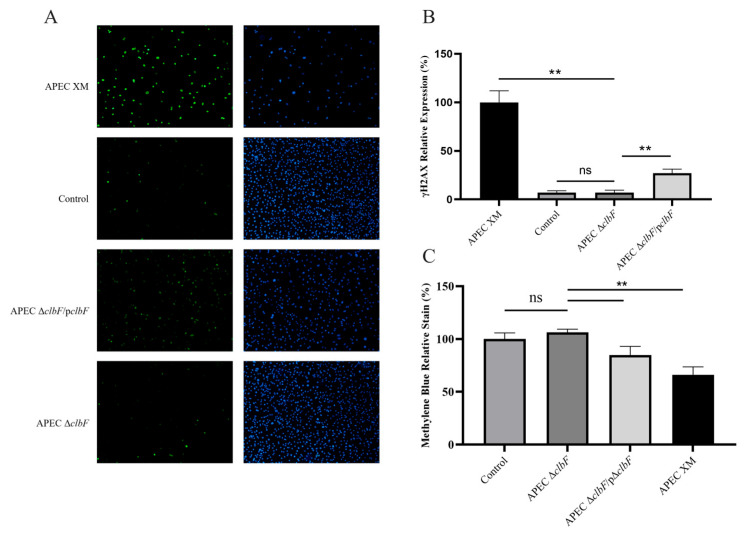
*ClbF* is essential for colibactin biosynthesis. (**A**) Nuclear DNA and γH2AX were stained blue and green, respectively. (**B**) Percentage of γH2AX-positive cells at 72 hpi, normalized to the APEC XM group (set as 100%). (**C**) Megalocytosis in methylene blue-stained cells was quantified by absorbance (600 nm), with control group data normalized to 100% (**, *p* < 0.01; ns, *p* > 0.05).

Colibactin production was assessed by quantifying γH2AX expression (a DSBs-sensitive marker) and megalocytosis in infected bEnd.3 cells. Significantly elevated γH2AX cell percentages were observed at 72 hpi in the WT and APEC Δ*clbF*/p*clbF* groups compared with the control group, whereas the APEC Δ*clbF* group showed no increase (see Figure 2A,B). Consistent with colibactin-induced cytopathic effects, the WT and APEC Δ*clbF*/p*clbF* strains caused progressive cellular enlargement (megalocytosis). Cytotoxicity quantification via methylene blue absorbance (600 nm) revealed significant reduction in these groups (Figure 2C), while APEC Δ*clbF*-infected cells maintained control-level absorbance.

### 3.3. clbF-Attenuated Virulence in In Vivo Infection

**Figure 3:**
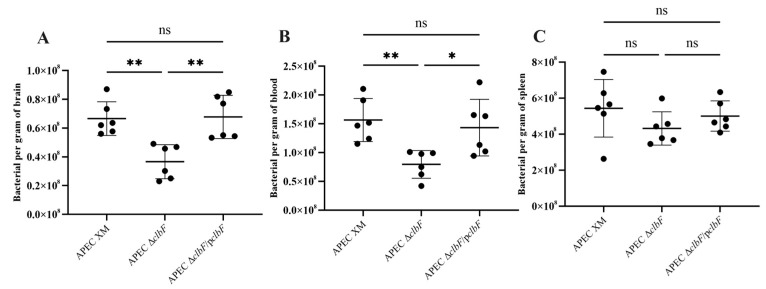
*ClbF* affects the pathogenicity of APEC XM. ICR mice were inoculated intraperitoneally with a dose of 10^7^ CFU bacteria or an equal volume of sterile saline. Bacterial load assay for the brain (**A**), blood (**B**), and spleen (**C**) by plate counting (**, *p* < 0.01; *, *p* < 0.05; ns, *p* > 0.05).

A mouse model was used to assess the virulence of wild-type, mutant, and complementation strains. The LD_50_ values of WT, APEC Δ*clbF*, and APEC Δ*clbF*/p*clbF* were 1.79 × 10^5^, 5.66 × 10^6^, and 3.16 × 10^5^ CFU/mouse, respectively (Table 1). There was a 3.16-fold decrease in virulence for mutant strain compared with the wild strain. Then, we tested the systemic infection effect of *clbF* in vivo. Bacterial loads in brain and blood at 24 hpi revealed significantly reduced colonization by APEC Δ*clbF* versus WT (Figure 3A,B). Complementation restored colonization capacity to WT levels across all organs. Thus, *clbF* deletion substantially diminished both virulence and systemic dissemination.

### 3.4. Expression of Virulence Factors

**Figure 4:**
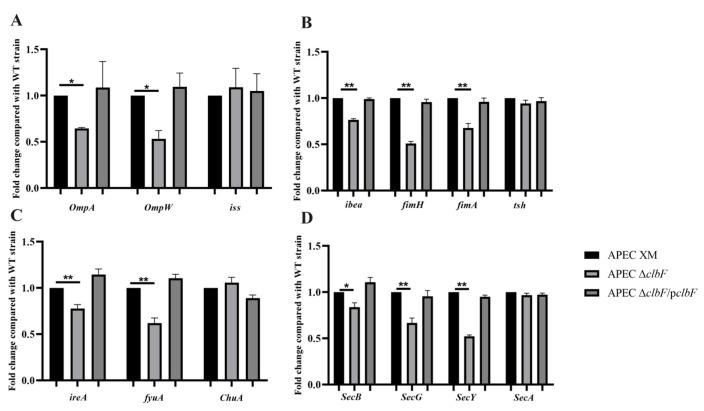
Related genes expression levels: (**A**) antiserum survival factor genes (*ompA*, *ompW*, and *iss*); (**B**) adherence and invasion genes (*ibea*, *fimH*, *tsh*, and *fimA*); (**C**) iron acquisition genes (*ireA*, *fyuA*, and *ChuA*); (**D**) secretion system genes (*Sec ABGY*). Transcript expression of the indicated genes was measured using RT-qPCR. *gapA* was used as the normalizing internal standard. Changes were calculated using APEC XM as the relative measure of comparison. (**, *p* < 0.01; *, *p* < 0.05).

RT-qPCR was used to quantify the expression of the antiserum survival factor genes (*ompA*, *ompW,* and *iss*), adherence and invasion genes (*ibea*, *fimH*, *tsh*, and *fimA*), iron acquisition genes (*ireA*, *fyuA*, and *ChuA*), and secretion system genes (*Sec ABGY*). The expressions of *ompA*, *ompW*, *iss*, *ibea*, *fimH*, *fimA*, *ireA*, *fyuA*, *Sec B*, *Sec G*, and *Sec Y* genes were down-regulated by 35.5%, 46.6%, 23.5%, 48.8%, 19.5%, 32.2%, 22.2%, 38.1%, 16.6%, 33.2%, and 47.6%, respectively, in the APEC Δ*clbF* when compared with their expression in the WT strain (Figure 4). There were no differences among all strains in the expression of *tsh*, *ChuA*, and *Sec A.* (Figure 4)

## 4. Discussion

Colibactin is a structurally uncharacterized genotoxin structure created by human gut commensal *Escherichia coli* strains containing a 54-kilobase nonribosomal peptide–polyketide biosynthetic gene cluster [5]. The *pks^+^* genotype predominates in *E. coli* phylogroups A, B1, B2, and D with significantly higher prevalence in B2 [23]. As most ExPEC strains belong to the highly pathogenic phylogroups B2 and D [24], this suggests that the presence of the *pks* island may be closely linked to the high pathogenicity of *Escherichia coli*. Notably, PEC and NMEC utilize similar pathogenic strategies for meningitis induction, positioning APEC as an emerging foodborne zoonotic pathogen and a potential reservoir for human extraintestinal infections. Consequently, APEC’s meningitis-causing mechanisms warrant urgent investigation. This study used a *pks*^+^ *E. coli* strain (isolated from a Muscovy duck with meningitis and sepsis), which has previously been used to induce meningitis in newborn or young avian and rodents [25,26]. Mechanistic studies indicate that *clbF* synthesizes the aminomalonyl (AM) extender unit essential for colibactin production [20,23]. However, *clbF*’s role in APEC pathogenicity remains undefined. Here, we constructed a *clbF*-deletion mutant to assess its impact on bacterial biology and virulence.

The γH2AX expression and methylene blue staining definitively demonstrated that *clbF* deletion severely impaired colibactin production, substantially reducing DNA damage caused by this genotoxin. Rapidly proliferating bacteria exhibit greater competitive advantage in host ecological niche colonization than their slower-growing counterparts. Growth test in vitro showed that *clbF* does not affect the growth ability of APEC XM. Both LD_50_ assays and the quantification of bacterial CFUs in 4-week infected mouse organs demonstrated significantly attenuated virulence in the *clbF*-deletion mutant. These findings establish colibactin as a critical virulence factor in APEC that mediates severe host pathology during infection.

*E. coli*-induced meningitis requires three essential steps: colonization/invasion, bacteremia, and blood–brain barrier (BBB) traversal. Intraperitoneal infection with *clbF*-deficient *E. coli* resulted in significantly reduced bacterial loads in the brains and blood of mice. We hypothesized that *clbF* deficiency may compromise bloodstream survival and expression of the BBB-crossing virulence factor. Based on prior dual RNA-seq findings [21], we therefore quantified the transcriptional expression of virulence genes, which were related to serum resistance, adhesion and invasion, the secretion system, and iron acquisition/metabolism.

Successful BBB traversal by pathogenic *E. coli* requires achieving sufficient bacteremia density in the bloodstream. In this study, we have tested three serum resistance-associated genes (*ompA*, *ompW* and iss). *OmpA*, encoding a 35 kDa protein essential for serum survival and blood–brain barrier invasion [27], additionally inhibits dendritic cell maturation by reducing the surface expression of costimulatory markers (such as CD40, HLA-DR, and CD86) [28]. *neuC* was predominantly associated with neonatal meningitis causing *E. coli* pathotype [29], which is involved in the biosynthesis of cell surface capsule [30]. In this study, we observed reduced bacterial loads in the blood of mice infected with the *clbF* deletion mutant, potentially linked to the downregulation of *ompA* and *neuC* expression.

Adhesive capacity is fundamental to APEC infectivity and dictates strain-specific pathogenicity variances. The ibeA locus orchestrates NMEC virulence factor expression and promotes early-onset neonatal meningitis pathogenesis through direct bacterial invasion and neutrophil transmigration [31,32,33]. As key virulence determinants, type 1 fimbriae mediate surface attachment and biofilm formation. Their biogenesis requires both FimH (receptor-specific adhesin) [34] and FimA (structural subunit) [35], which facilitate initial phagocytosis while conferring protection against heterophil killing [36]. Separately, *tsh* gene encodes a proteolytic autotransporter protein that adheres to red blood cells, hemoglobin, and extracellular matrix components [37]. In this study, we observed significant downregulation of major pilus components and *ibeA* but no detectable change in *tsh* expression. This likely accounts for the discrepancy between the unchanged adhesion/invasion capacity in vitro and the impaired BBB traversal in the mouse infection model.

Iron is essential for *E. coli* growth and pathogenesis, driving diverse acquisition strategies [38,39]. The ferric yersiniabactin transporter *fyuA* enables bloodstream survival [40] and correlates with B2 phylogroup prevalence [41]. In human ExPEC, the virulence factor i*reA* mediates iron uptake while enhancing adhesion/stress resistance across hosts [38,42]. Although *ChuA* (a heme-iron receptor) is widely distributed [43], it is non-essential for bacteremia or adhesion/invasion [44]. In this study, we found that the expression of *fuyA* and *ireA* was downregulated in the *clbF*-deletion mutant, potentially explaining the significantly reduced infection rate of the ICR mice. However, the specific underlying mechanisms require further investigation.

While ClbM (a MATE transporter of colibactin) is essential for pre-colibactin translocation to the periplasm [45], its precise mechanism remains elusive. Our dual RNA-seq of TW infecting host cells revealed upregulation of general secretion (Sec) system genes [21]. The Sec system comprises *SecA*, *SecB*, and two complexes, *SecYEG* and *SecDFYajC* [46], which transports virulence factors across membranes and supports pathogenesis [47]. In this study, we found that the deletion of *clbF* results in decreased transcription levels of *SecB*, *ecG*, and *SecY* but does not affect *SecA* transcription. The general system is closely linked to the transport of multiple virulence factors of *E. coli*. A significant reduction in mortality was observed in the APEC Δ*clbF* group, which is likely also attributable to the diminished of colibactin production. However, the precise mechanisms involved need further investigation.

## 5. Conclusions

Our experiments demonstrated that the deletion of *clbF* blocks colibactin production. Despite having no in vitro effect on adhesion or invasion, the loss of *clbF* reduced bacterial pathogenicity, an effect potentially associated with genes involved in survival, adhesion/invasion, iron uptake, and secretion systems. These results establish *clbF* as a key virulence factor essential for APEC XM to induce meningitis.

## Figures and Tables

**Table 1:** Calculations of LD50.

Dose of Challenge (CFU)	No. of Dead Mice		
	APEC XM	APEC Δ*clbF*	APEC Δ*clbF*/p*clbF*
1 × 10^7^	5/5	3/5	4/5
1 × 10^6^	4/5	1/5	4/5
1 × 10^5^	1/5	1/5	1/5
1 × 10^4^	1/5	0/5	1/5
LD_50_	1.79 × 10^5^	5.66 × 10^6^	3.16 × 10^5^

## Data Availability

Dataset available on request from the authors.

## Supplementary Materials

The following supporting information can be downloaded at https://www.mdpi.com/article/10.3390/vetsci12080727/s1, Table S1: Summary of bacterial strains, plasmids, and primers used in this study; Figure S1: the original image of Figure 1A.
