# Supplementary material for: The Role of clbF in the Pathogenicity of Avian Pathogenic Escherichia coli
Source: Vet Sci. 2025 Aug 1;12(8):727. doi: 10.3390/vetsci12080727 (PMC12390127; doi:10.3390/vetsci12080727)
Supplement: Supplementary file 1 [file vetsci-12-00727-s001.zip › vetsci-3735092-supplementary.pdf]

Table S1. Summary of bacterial strains, plasmids, and primers used in this study

Strains, Plasmids, or primers	Characteristic or Function	Source
Strains		
Avian pathogenic *Escherichia coli* (APEC) XM	Virulent strain of APEC	Donated by Dr. Guoqiang Zhu, Yangzhou University
APEC XM Δ*clbF*	Deletion mutant of *clbF* with APEC XM background	This study
APEC XM Δ*clbF*/p*clbF*	APEC-XM Δ*clbF* with the vector pACYC184-*clbF*, Cm^r	This study
**Plasmid**		
pKD46	λ red recombinase expression plasmid	
pKD3	pANTSγ derivative containing FRT-flanked, Cm^r	
pCP20	temperature-sensitive replication and thermal induction of FLP synthesis	
pBR322-*clbF*	pBR322 containing the promoter followed by the full-length *clbF*, Cm^r	This study
	Sequence (5′→3′)	Product size
P1	ACATACTTCGCCATCAGGA	900/251
P2	CCAAACAACATCTCGCCC	
P3	GTATTCCGCCGCGCTGAAGCACATTAGTTGCGCACTGCG CGTTTGGGTCAGCATTGTGTGTGTAGGCTGGAGCTGCTTCG	750bp
P4	TCGCCCTGGCTGCACAGAATATTGAACGTCAGCAGTTTA TTGTGCCAGCATATGAATATCCTCCTTAG	
P5	TAACGCAGTCAGGCACCGTGTCTGGAGTAAACTATG TGCACTG	900
P6	GTGAATCCGTTAGCGAGGTGCCCATCCTTCGTCATG CTTCACT	
gapA-F	CGTTAAAGGCGCTAACTTCG	
gapA-R	ACGGTGGTCATCAGACCTTC	
iss-F	CGGGAATTGGACAAGAGAAAAC	
iss-R	TTTCTGCACCGCCACAAA	
ompA-F	TGGGTGTTTCCTACCGTTTC	
ompA-R	GAGTGAAGTGCTTGGTCTGT	
ompW-F	ACCGTTCATCTGCCACCAAC	
ompW-R	GCCCTGCCTCTTTGCCATGATC	
ibeA-F	AGGCAGGTGTGCGCCGCGTAC	
ibeA-R	TGGTGCTCCGGCAAACCATGC	
fimH-F	CTTATGGCGGCGTGTTATCT	
fimH-R	CGGCTTATCCGTTCTCGAATTA	
fimA-F	FGCACAGGAAGGAGCAACCA	
fimA-R	GGCAACAGCGGCTTTAGATG	

tsh-F	CACGGGTTGTGGGATTCAG
tsh-R	TTACGTGCAACCTGGTAACCAT
ireA-F	AAACATGGGATGGCGTACTT
ireA-R	AATCAATGGGCCTGACAGATAG
fyuA-F	ATGCCTATGTGGGATGGAATG
fyuA-R	CCAGTCATCGGTGGTGTATTT
ChuA-F	TAGGCCACATCAAGGCTAAAC
ChuA-R	CGGCGACAACTATGTCGTATAA
secY-F	CGCTGTTGCTCAAGCAGTTT
secY-R	TATCGGTGCGCTGATTGTGT
secF-F	ATGCATTGCAAAAAGCCGGT
secF-R	CGGCATACGGACCATGATGT
secB-F	GGCAATTGGCCTTGTTGGTC
secB-F	AACCAAACAGCGTAGCGGAA
secG-F	TGTGGCAATTGGCCTTGTTG
secG-R	CTCCGAAGGAGGCTCCCATA

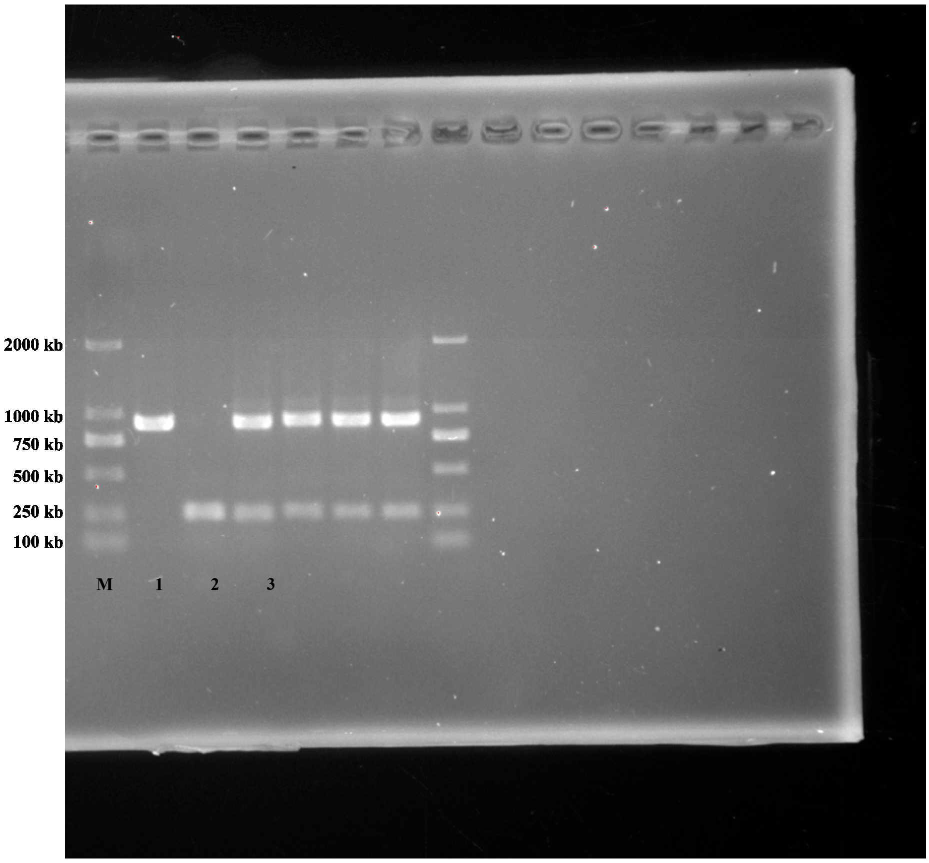

Figure S1. the original image of Figure 1A
